# European *Aedes caspius* mosquitoes are experimentally unable to transmit Zika virus

**DOI:** 10.1186/s13071-019-3620-7

**Published:** 2019-07-25

**Authors:** Ana I. Núñez, Sandra Talavera, Carles Aranda, Lotty Birnberg, Raquel Rivas, Núria Pujol, Marta Verdún, Anna-Bella Failloux, Núria Busquets

**Affiliations:** 1 Institut de Recerca i Tecnologia Agroalimentàries (IRTA), Centre de Recerca en Sanitat Animal (CReSA), Campus de la Universitat Autònoma de Barcelona, Bellaterra (Cerdanyola del Vallès), 08193 Barcelona, Spain; 2 Servei de Control de Mosquits, Consell Comarcal del Baix Llobregat, Barcelona, Spain; 3 Department of Virology, Arboviruses and Insect Vectors Unit, Institut Pasteur, Paris, France

**Keywords:** *Aedes caspius*, Saliva, Transmission, Arbovirus, Vector competence, Zika virus

## Abstract

**Background:**

*Aedes caspius* (Pallas, 1771) is a floodwater mosquito species widely distributed in the Western Palaearctic. As an anthropophilic species, its role as an arbovirus vector may be the key for understanding the transmission cycle of certain diseases in Europe such as Zika virus (ZIKV). Concerning vector competence for ZIKV, studies related to *Ae. caspius* are still scarce. ZIKV is an arbovirus that has provoked a widespread epidemic in the Pacific region (2007–2013) and in the Americas (2015–2016). ZIKV is associated with serious neurological injuries (e.g. microcephaly) and Guillain-Barré syndrome. Due to the ZIKV epidemics in the American continent, some viraemic travellers coming from endemic countries have been reported in Europe. More knowledge is therefore required to define the susceptibility of autochthonous mosquito species such as *Ae. caspius* for ZIKV in order to improve arbovirus surveillance and control programmes. In the present study, the vector competence of a European population of *Ae. caspius* was evaluated for two ZIKV lineages, the Suriname ZIKV strain (Asian lineage) and the MR766 ZIKV strain (African I lineage). Females were tested at 7, 14 and 21 days post-exposure (dpe) to infectious blood meals. An *Ae. aegypti* PAEA strain was used as a positive control.

**Results:**

*Aedes caspius* presented low susceptibility to ZIKV infection and the virus was only detected by RT-qPCR in body samples. Low viral loads were detected for the MR766 strain at 7 dpe and for the Suriname strain at 14 and 21 dpe. *Aedes caspius* was unable to produce a disseminated infection and virus transmission at any of the tested time points. Using *Ae. aegypti* PAEA strain, infection, dissemination and transmission rates were calculated for the Suriname ZIKV strain (Asian lineage) at each time point. For the MR766 ZIKV strain (African I lineage), while only infection rates were estimated at each time point, no dissemination or transmission were detected in either species.

**Conclusions:**

The results of the present study reveal that the tested *Ae. caspius* population has a strong midgut escape barrier that limits the dissemination or transmission of the virus. As such, it seems unlikely that European *Ae. caspius* mosquitoes could be involved in ZIKV transmission if ZIKV was introduced into Europe. This information may help in designing a better strategy to European surveillance and control programmes for ZIKV.

## Background

*Aedes caspius* (Pallas, 1771) is a floodwater mosquito species widely distributed in the Western Palaearctic [1]. This mosquito species is tolerant to varying levels of salinity in larval breeding places [2] and so is present in different habitats, including coastlands, irrigation channels, swamps and rice fields. *Aedes caspius* is an anthropophilic species and a crepuscular feeder and is known to bite during the day and night [3]. These mosquitoes usually feed aggressively on humans and animals, both indoors and outdoors [4]. Thus its role as an arbovirus vector may be the key for the knowledge of transmission cycle of certain diseases in Europe, especially given its anthropophilic behavior. The vector competence of some *Ae. caspius* populations exposed to different arboviruses has been tested in previous studies. *Aedes caspius* populations from the Camargue (France) were experimentally found to be potential vectors of arboviruses such as chikungunya virus (CHIKV) [5] and Rift Valley fever phlebovirus (RVFV) [6]. However, *Ae. caspius* from Camargue and from Andalusia (Spain) were unable to transmit West Nile virus (WNV) [7] and Zika virus (ZIKV) [8], respectively.

The ZIKV is an arthropod-borne virus belonging to the genus *Flavivirus* (family *Flaviviridae*). The virus is primarily transmitted in a zoonotic cycle between mosquitoes and non-human primates in Africa, although sexual [9] and perinatal [10] ZIKV transmission have also been confirmed in humans. The virus has been associated with severe clinical manifestations and congenital malformations including microcephaly [11] and Guillain-Barré syndrome [12]. ZIKV was isolated for the first time from a rhesus macaque monkey in the Zika forest (Uganda) in 1947 [13]. The virus was subsequently found in Asia in the 1960s. In 2007 there was an outbreak of ZIKV in Yap Island, Micronesia [14], which spread to Pacific islands in 2013 [15] before reaching Latin America in 2015 [16]. Nowadays, all isolated ZIKVs are grouped into three lineages: Asian; African I; and West African II [17, 18]. ZIKV has been isolated from numerous African mosquito species in field [19] but *Aedes aegypti* is considered to be the main vector of ZIKV in urban areas [20]. In addition, several *Aedes* species from all continents have been observed to transmit the virus experimentally: e.g. *Aedes vittatus*, *Aedes vexans* [20], *Aedes polynesiensis* [21] and *Ae. albopictus* [22–26].

Due to the ZIKV epidemics in the American continent, some viraemic travellers coming from endemic countries have been reported in Europe [27], especially during the summer months [28], raising important alarms for human health. In this context, more in-depth knowledge is required about the susceptibility of autochthonous mosquito species such as *Ae. caspius* for ZIKV to improve arbovirus surveillance and control programmes. For this reason, in the present study we evaluated the vector competence of an *Ae. caspius* mosquito population from El Prat de Llobregat (Catalonia, Spain) for two ZIKV lineages, Suriname (Asian lineage) and MR766 (African I lineage) to measure its potential role in ZIKV transmission.

## Methods

### Mosquito rearing

*Aedes caspius* larvae were collected from El Prat de Llobregat (Catalonia, Spain) in October 2017. Larvae were reared in trays containing dechlorinated water supplemented with fish food (Goldfish, Tetra GmbH, Melle, Germany) until the adult stage. Emerging adults were maintained at 26/22 °C (day/night) to simulate summer environmental conditions at the latitude where the mosquitoes were captured, a relative humidity of 80% and a light/dark photocycle of 14:10 h. Mosquitoes were fed *ad libitum* with 10% sucrose solution. The F0 generation was used for experimental infection. An *Ae. aegypti* PAEA strain, from Paea (Tahiti, French Polynesia), colonized since 1994, was reared in the same conditions and used as a positive control of ZIKV vector.

### Virus production and titration

Suriname and African MR766 ZIKV strains provided by the European EVAg project were used in the present work. The Suriname ZIKV strain (EVAg no. 011V-01621; Asian lineage) was isolated from a placental material of a patient in Netherlands in 2016 who came from Suriname during the ZIKV epidemic [29] and the African MR766 ZIKV strain (African I lineage) was isolated from a rhesus monkey (*Macaca mulatta*) in 1947 in Uganda [13]. These strains were propagated and titrated to obtain the 50% tissue culture infective dose per ml (TCID_50_/ml) in African green monkey kidney (VERO) cells.

### Experimental infection of mosquitoes

Forty-eight hours before exposure of mosquitoes to the infectious blood meal, the 10% sucrose solution was removed to increase mosquito appetite. Totals of 480 *Ae. caspius* and 275 *Ae. aegypti* females (7–10 days old) were exposed to infectious blood at 7 log_10_ TCDI_50_/ml of both Suriname and MR766 strains and 20 females of each species were exposed to non-infectious blood. For blood-meal preparation, rabbit blood was washed and mixed with adenosine 5’-triphosphate (ATP) (5 × 10^−3^ M) (Sigma-Aldrich, St. Louis, MO, USA) and virus (infectious) or cell culture medium (DMEM) (non-infectious). Females were exposed to a Hemotek^®^ artificial feeding system (Discovery Workshop, Accrington, UK) at 37.5 ± 0.5 °C for 30 min. After exposure, females were anesthetized with CO_2_. Fully engorged females were selected and maintained in groups of ten in cardboard cages (Watkins & Doncaster, Leominster, UK) under the same rearing environmental conditions. Throughout the experiment, the mosquitoes were maintained with permanent access to 10% sucrose solution in cotton pledges. After a period of 7, 14 and 21 days post-exposure (dpe) to infectious blood, females were anesthetized with CO_2_, legs and wings were dissected and saliva was extracted using the capillary technique as previously described [30]. The number of mosquitoes tested at each time point is summarized in Table 1. Body, leg and wing samples were stored in 0.5 ml of Dulbecco’s modified Eagle’s medium (DMEM) (Lonza Group AG, Basel, Switzerland) and the saliva samples in 0.190 ml of DMEM at − 80 °C until ZIKV detection. Infection (IR), disseminated infection (DIR) and transmission rates (TR) were estimated to evaluate the vector competence [31]. The IR represents virus replication in the midgut epithelial cells. The DIR shows that the virus was able to cross the midgut barrier and reach the hemocoel. The TR shows that the virus was able to cross the salivary glands barrier. We also measured the transmission efficiency (TE), which refers to the rate of mosquitoes with infectious saliva among the total mosquitoes assayed. The experimental infection was performed at IRTA, CReSA BLS3 facilities.

**Table 1:** Summary of assays

Mosquito species tested	ZIKV strain tested	Titer of ZIKV (TCID_50_/ml)	No. of mosquitoes tested per time point			
			7 dpe	14 dpe	21 dpe	Total
*Ae. caspius*	Suriname	7 log_10_ TCID_50_/ml	30	30	30	90
	MR766	7 log_10_ TCID_50_/ml	30	30	30	90
	Negative control	No virus	–	–	8	8
*Ae. aegypti*	Suriname	7 log_10_ TCID_50_/ml	20	20	20	60
	MR766	7 log_10_ TCID_50_/ml	20	20	19	59
	Negative control	No virus	–	–	11	11

*Abbreviation*: dpe, days post-exposure

### ZIKV detection

Virus detection from leg, wing and body samples was carried out using 1/10 and 1/100 dilutions in 96-well plates containing a Vero cell monolayer. Saliva samples were titrated directly in 96-well plates in a Vero cell monolayer. Vero cells were maintained with DMEM supplemented with 2% FCS and 2% of penicillin/streptomycin/nystatin (1000 U/ml, 10 mg/ml and 500 U/ml, respectively; Sigma-Aldrich) and incubated for seven days at 37 °C and 5% CO_2_ until cytopathic effect observation.

Prior to viral RNA extraction, the samples were homogenized using a TissueLyser II (Qiagen GmbH, Hilden, Germany) at 30 Hz for 1 min. Viral RNA was extracted from the samples using NucleoSpin^®^ RNA Virus (Macherey-Nagel, Düren, Germany) according to the manufacturer’s protocol. Zika RNA was detected by reverse-transcription quantitative PCR (RT-qPCR) using the primers ZIKA 1086 and ZIKA 1162c defined previously [32] and AgPath-ID™ One-Step RT-PCR reagents (Applied Biosystems, Foster City, CA, USA). The nucleic acids were detected with a Real-Time PCR 7500 Fast System (Applied Biosystems) with the following amplification protocol: 45 °C for 10 min; 95 °C for 10 min; then 45 cycles at 95 °C for 15 s and at 60 °C for 45 s. The RT-qPCR sensibility was 0.451 TCID_50_/reaction for detection of MR766 and 0.035 TCID_50_/reaction for Suriname ZIKV strains.

## Results

Feeding rates were 56% (280/500) and 77.45% (213/275) for *Ae. caspius* and *Ae. aegypti*, respectively.

**Fig. 1:**
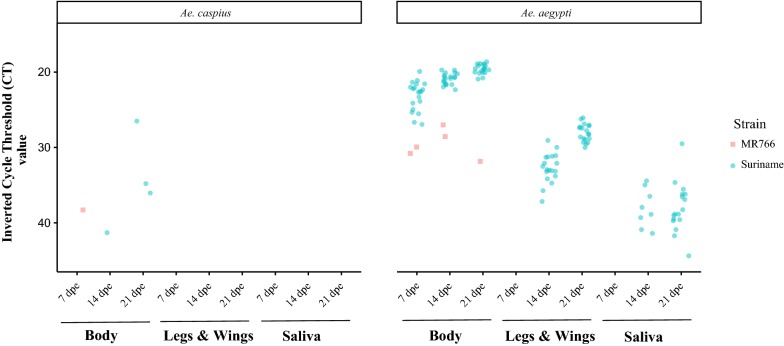
Results of the RT-qPCR for both mosquito species tested. The CT values are blue for the Suriname strain and red for MR766

For the *Ae. caspius* population, ZIKV was detected by RT-qPCR only in body samples for both virus strains used in the present study (Fig. 1). As detailed in Table 2, the infection rate (IR) in *Ae. caspius* for the Suriname ZIKV strain was 3.33% and 10% at 14 and 21 days post-exposure (dpe), respectively. For the MR766 ZIKV strain, the IR was only 3.33% at 7 dpe. In addition, both ZIKV strains were unable to induce a disseminated infection and transmission in *Ae. caspius*.

**Table 2:** Infection, disseminated infection and transmission rates of *Ae. caspius* from Catalonia and *Ae. aegypti* PAEA populations artificially fed with Suriname and MR766 ZIKV strains at a titer of 7 log_10_ TCID_50_/ml. Viral detection was performed by the visualization of cytopathic effect on cells and RT-qPCR assays

Dpe	Species	Suriname						MR766					
		IR (%)		DIR (%)		TR (%)		IR (%)		DIR (%)		TR (%)	
		CE	RT-qPCR	CE	RT-qPCR	CE	RT-qPCR	CE	RT-qPCR	CE	RT-qPCR	CE	RT-qPCR
7	*Ae. caspius*	0/30 (0)	0/30 (0)	0/0 (0)	0/0 (0)	0/0 (0)	0/0 (0)	0/30 (0)	1/30 (3.33)	0/0 (0)	0/1 (0)	0/0 (0)	0/0 (0)
	*Ae. aegypti*	17/20 (85)	20/20 (100)	0/17 (0)	0/20 (0)	0/0 (0)	0/0 (0)	2/20 (10)	2/20 (10)	0/2 (0)	0/2 (0)	0/0 (0)	0/0 (0)
14	*Ae. caspius*	0/30 (0)	1/30 (3.33)	0/0 (0)	0/1 (0)	0/0 (0)	0/0 (0)	0/30 (0)	0/30 (0)	0/0 (0)	0/0 (0)	0/0 (0)	0/0 (0)
	*Ae. aegypti*	20/20 (100)	20/20 (100)	9/20 (45)	19/20 (95)	3/9 (33.3)	8/19 (52.6)	2/20 (10)	2/20 (10)	0/2 (0)	0/2 (0)	0/0 (0)	0/0 (0)
21	*Ae. caspius*	0/30 (0)	3/30 (10)	0/0 (0)	0/3 (0)	0/0 (0)	0/0 (0)	0/30 (0)	0/30 (0)	0/0 (0)	0/0 (0)	0/0 (0)	0/0 (0)
	*Ae. aegypti*	19/20 (95)	19/20 (95)	16/19 (84.2)	19/19 (100)	1/16 (6.2)	17/19 (89.4)	1/19 (5.2)	1/19 (5.2)	0/1 (0)	0/1 (0)	0/0 (0)	0/0 (0)

*Abbreviations*: CE, cytopathic effect; IR, infection rate; DIR, disseminated infection rate; TR, transmission rate; dpe, days post-exposure

*Note*: Data are given as number of positive/number of tested samples (percentage)

In our positive control (*Ae. aegypti* PAEA strain), infection was detected at 7 dpe for both ZIKV strains tested by RT-qPCR and cytopathic effect while dissemination and transmission were only found for the Suriname ZIKV strain at 14 and 21 dpe (Table 1 and Fig. 1). The transmission efficiency (TE) of *Ae. aegypti* for the Suriname ZIKV strain based on the cytopathic effect in Vero cells was 15% (3/20) and 5% (1/20) at 14 and 21 dpe, respectively.

## Discussion

The present study demonstrates that the assessed population of *Ae. caspius* from Catalonia was unable to transmit the Suriname and MR766 ZIKV strains, belonging to the Asian and African I phylogenetic lineages, respectively. Our results indicate that the *Ae. caspius* population has a strong midgut escape barrier (MEB) since at 7 dpe we found infected bodies of MR766 ZIKV strain by RT-qPCR as well as at 14 and 21 dpe to the ZIKV Asian strain but neither the disseminated infection nor the transmission were detected at any of the time points analyzed. These findings are in agreement with the hypothesis that the MEB can limit virus dissemination from the midgut to the hemocoel or secondary organs as reported for other arbovirus-mosquito species combinations [33, 34]. As the viral load detected by RT-qPCR was low in infected bodies (Fig. 1), we suggest that the virus is replicating at a very low level. Therefore, the virus would not be able to cross the MEB and disseminate through the mosquito hemocoel to reach the salivary glands making it unable to transmit the virus. In addition, these results are in accordance with a recent study in which another *Ae. caspius* population was assayed for a ZIKV strain of the Asian lineage (Puerto Rico, 2015) under constant environmental conditions [8]. It should be noted that in our experiment, apart from testing a ZIKV strain of Asian lineage, we also assayed a ZIKV strain of the African I lineage (MR766) with cycled environmental conditions (26/22 °C day/night). Both studies indicate that despite the anthropophilic behavior of *Ae. caspius*, its role in the transmission of ZIKV seems unlikely.

*Aedes aegypti* from PAEA (French Polynesia), selected as a positive control, was able to transmit the Suriname strain (Asian lineage). Suriname ZIKV infection, dissemination and transmission were observed at 14 and 21 dpe. These results are in agreement with other experiments reported on *Ae. aegypti* vector competence for ZIKV [35, 36]. However, the MR766 strain (African I lineage; the historical strain isolated in 1947 in Uganda [13]) was unable to disseminate and be transmitted in this mosquito species. This ZIKV strain is an old strain that has suffered several passages in mice and cells from various laboratory sources that we assume may have influenced the vector competence assays in both assayed mosquito species. Furthermore, the inefficient dissemination and transmission in *Ae. aegypti* (PAEA strain) exposed to the MR766 ZIKV strain were in agreement with previous results reported by Diagne et al. [37]. The differences observed between our results and other vector competence experiments in which dissemination and transmission of the MR766 ZIKV strain were reported for *Ae. aegypti* [26, 35, 36, 38, 39], could be explained by the genetic variability of *Ae. aegypti* populations as mentioned by Diagne et al. [37]. Furthermore, it is known that temperature can influence vector competence as described for several mosquito species infected with other arboviruses (e.g. dengue virus (DENV), CHIKV [40] or WNV [41–43]). A recent study showed that temperature may directly affect vector competence for ZIKV rendering the *Ae. aegypti* population tested at low temperature (18 °C) unable to transmit the virus [44]. Therefore, the environmental conditions used in the present study could have also influenced our results; in earlier studies where higher dissemination and transmission were observed in the *Ae. aegypti*-MR766 ZIKV pairing, the assays were performed at a temperature of 28 °C in contrast to the 26/22 °C (day/night) used in our experiment (which we used to mimic summer environmental conditions in the area where the *Ae. caspius* population was captured).

Finally, with respect to the techniques used for virus detection in our vector competence assays, the RT-qPCR results had a slightly better sensitivity than those obtained by cytopathic effect in all mosquito-virus pairings. However, although RT-qPCR was more sensitive, the cytopathic effect caused by the virus allows better knowledge of its viability which is more useful for a better estimation of the transmission efficiency of a mosquito population. Therefore, we strongly recommend that for the vector competence studies viable viruses in the saliva should be taken in to account to determine the transmission efficacy.

## Conclusions

Given the high risk of ZIKV introduction in Europe *via* infected travelers coming from endemic areas, it is important to know if anthropophilic European mosquito populations are able to transmit this virus and sustain a ZIKV outbreak. Our results indicate that it is unlikely that *Ae. caspius* mosquitoes from Spain, particularly from Catalonia, could be involved in the transmission of ZIKV if it was introduced. Therefore, *Ae. caspius* is not a relevant species to be monitored and controlled in case of ZIKV introduction. This is useful and crucial information for the health authorities with respect to the establishment of efficient surveillance and vector control programmes for ZIKV. Moreover, our study highlights the importance of performing vector competence assays for each arbovirus-vector mosquito species in specific environmental conditions to provide information for more accurate predictions of the risk of arbovirus transmission in a specific area.

## Data Availability

All data generated or analyzed during this study are included in this published article.

## Abbreviations

ZIKV: Zika virus
dpe: days post exposure
IR: infection rate
DIR: disseminated infection rate
TR: transmission rate
TE: transmission efficiency
MEB: midgut escape barrier
CHIKV: chikungunya virus
DENV: dengue virus
RVFV: Rift Valley fever phlebovirus
